# Supplementary material for: The interactome of intact mitochondria by cross-linking mass spectrometry provides evidence for coexisting respiratory supercomplexes
Source: Mol Cell Proteomics. 2017 Dec 8;17(2):216–32. doi: 10.1074/mcp.RA117.000470 (PMC5795388; doi:10.1074/mcp.RA117.000470)
Supplement: Supplemental Data [file supp_RA117.000470_133922_0_supp_23978_fzffwf.zip › spectra_annotation/mito_DR_spectra_annotation/451-1-88-1-2-1.pdf]

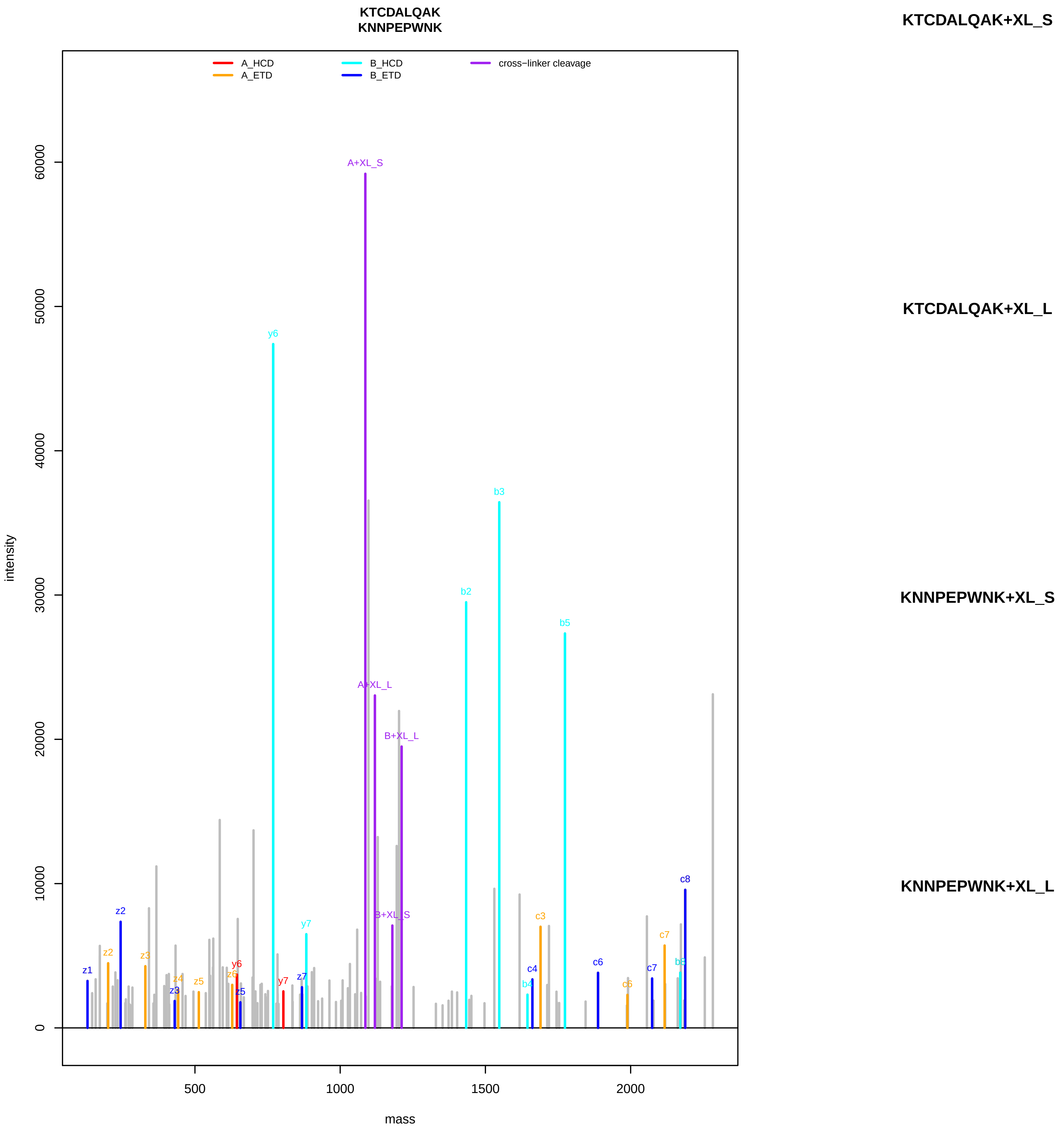
KTCDALQAK
KNNPEPWNK
A_HCD
A_ETD
B_HCD
B_ETD
cross−linker cleavage
intensity
mass
0
10000
20000
30000
40000
50000
60000
500
1000
1500
2000
A+XL_S
A+XL_L
B+XL_L
B+XL_S
y6
y7
b2
b3
b4
b5
b6
z1
z2
z3
z4
z5
z6
z7
c3
c4
c6
c7
c8
KTCDALQAK+XL_S
KTCDALQAK+XL_L
KNNPEPWNK+XL_S
KNNPEPWNK+XL_L
